# Relationship between FDI, fiscal expenditure and green total-factor productivity in China: From the perspective of spatial spillover

**DOI:** 10.1371/journal.pone.0250798

**Published:** 2021-04-30

**Authors:** Ke-Liang Wang, Shuang He, Fu-Qin Zhang

**Affiliations:** School of Economics, Ocean University of China, Qingdao, PR China; China University of Mining and Technology, CHINA

## Abstract

Deeply investigating the relationship between foreign direct investment (FDI), fiscal expenditure and green total-factor productivity (GTFP) is beneficial to formulating effective policies to promote the high-quality development in China. Based on theoretical mechanism analysis, with panel data of China’s mainland 30 provinces during 2003–2017, this paper utilizes spatial econometric model to empirically explore the effects of FDI, fiscal expenditure and their interaction item on the growth of GTFP in China. The results show that FDI significantly promote the growth of the local and its neighboring GTFP, and both fiscal expenditure and the interaction between FDI and fiscal expenditure exert significantly negative effects on the growth of GTFP in the local and its neighboring regions. A series of robustness checks and the endogeneity test can ensure the reliability of these results. In addition, great heterogeneity can be found across China’s different regions in the relationship between FDI, fiscal expenditure and GTFP. The conclusions suggest that it is necessary to give fully play to the synergy between FDI and fiscal expenditure and formulate regionally targeted policies to improve GTFP and promote high-quality development in China.

## 1. Introduction

Since the reform and opening up in 1978, China’s economy has experienced rapid growth and currently become the world’s second largest economy. However, the rapid economic growth has also brought about increasingly severe resource and environmental problems [1,2]. At present, China’s energy consumption and carbon dioxide emissions have ranked first in the world, and the emissions of major pollutants are also among the world’s forefront [3,4]. Based on the data disclosed in the “State of the Environment Bulletin in China in 2018”, there were 217 cities in China did not meet the air quality standard in 2018, accounting for 64.2% of the country’s total, fully demonstrating the incoordination between economic development and environmental protection in China. The Chinese government has realized the importance of resource conservation and environmental protection and has issued a series of effective measures to address it. Currently, vigorously promoting green development and achieving the transformation of the traditional economic development mode have been given priority in China and has become one of the country’s national strategy [5,6]. The Chinese government clearly stated that sacrificing the ecological environment in exchange for short-term economic growth is not be accepted and further proposed the new development concept of “lucid waters and lush mountains are invaluable assets”, highlighting the importance of environmental protection in the process of economic development [7]. Then, how to put the new development concept into implementation and substantially promote the high-quality development in China? For this question, numerous scholars have verified that green total-factor productivity (GTFP) improvement is a key engine [8,9]. Therefore, promoting GTFP has important theoretical value and practical significance for China to coordinate economic growth, resources utilization and environmental protection as well as achieve sustainable development [10,11].

Economic and institutional factors are respectively the external and internal factors affecting GTFP for an economy. Since the resources and environment have the attributes of public goods, the market economy cannot solve the problems of energy consumption and ecological pollution. Therefore, it is necessary to introduce government tools other than the market mechanism as a supplement, and there is also a close inner relationship between market economy and government tools [12]. Thus, it is necessary to incorporate them into a unified analytical framework when exploring the influencing factors of GTFP. In light of this, the research interest of this paper is to focus on the impact of foreign direct investment (FDI) as an economic factor and fiscal expenditure as an institutional factor and their interaction on China’s GTFP. Specifically, on the one hand, it is well known that FDI has always been an important driving force for China’s rapid economic growth, and its scale expansion and quality enhancement have made considerable contributions to China’s economic development since 1978 [13–15]. On the other hand, fiscal policies are important government’s macro-control means, in which fiscal expenditure is the dominant and its structure has been demonstrated to play profound impacts on TFP and environmental protection [16,17] With the deepening of economic globalization and the continuous deterioration of environmental quality, the linkage mechanism between FDI and government fiscal expenditure has become more and more complicated. Thus, in order to effectively promote China’s GTFP growth, this paper combines FDI, fiscal expenditure and GTFP into a unified analytical framework, and deeply investigates the relationship between them from both theoretical and empirical perspectives, with the aim to provide more valuable guidance and reference for related policy-making.

This paper attempts to contribute to existing literature in the following two aspects. First, Existing literature analyzes the impact on GTFP from the perspective of FDI and fiscal expenditures, and does not take into account the interaction between FDI and fiscal expenditures. Thus, the conclusions drawn from a single perspective have limitations. This paper incorporates FDI, fiscal expenditure and GTFP into a unified framework, and maybe it is the first time to explore the impact of the interaction between FDI and fiscal expenditure on GTFP, providing a more comprehensive analysis and significant guidance for China to improve GTFP. Second, different from existing studies, this paper will concentrate on investigating the direct effects and indirect effects of FDI and fiscal expenditure on China’s GTFP from spatial spillover perspective, and provide a new perspective for GTFP research, while considering the current situation of unbalanced and insufficient regional development. Meanwhile, this paper also takes into account the current situation of insufficient regional development imbalance, providing more insightful information for the improvement of GTFP in China.

The rest of this paper can be arranged as follows. Section 2 reviews related literature. Section 3 offers theoretical analysis and proposes research hypothesis. Section 4 conducts model specification and data description. Section 5 presents the results of empirical analysis. Section 6 concludes this paper with some policy implications.

## 2. Literature review

In recent years, the relevant literature on GTFP is considerable abundant, which can be roughly divided into two categories, one is the measurement of GTFP, the other is the influencing factors of GTFP. Regarding the approach of GTFP measurement, since Chung et al. (1997) first proposed Malmquist-Luenberger (ML) productivity index derived from directional distance function (DDF) to measure environmental total-factor productivity (TFP) [18], the ML index and its extended forms have been widely applied to measure the GTFP for different economic entities, which includes conventional ML index [8,9], Global ML (GML) index [19–21], Meta-frontier ML (MML) index [22–24], Biennial ML (BML) index [25,26] and Luenberger indicator [27]. With respect to the influencing factors of GTFP, they could be classified into economic and institutional factors. Among them, economic factors include technical change [28,29], industrial agglomeration [30], financial development [31,32], FDI [13,33,34]; institutional factors include environmental policies [35–38], fiscal policies [17,39]. Given the research theme of this paper, we mainly focus on and review the related studies on how FDI and fiscal expenditure affect GTFP growth.

A great number of previous studies have demonstrated that FDI is an important factor affecting TFP and environmental quality [40,41]. With regard to the relationship between FDI and environmental quality, related studies hold two opposing viewpoints, namely the “pollution heaven” and “pollution halo” hypothesis, respectively. Specifically, Walter and Ugelow (1979) first proposed the hypothesis of “pollution heaven” and believed that facing stricter environmental regulations in their own countries, developed countries’ enterprises are inclined to transfer their pollution-intensive industries to developing countries through FDI, which leads to environmental quality deterioration in developing countries [42]. Many scholars have verified this hypothesis. For instance, Cole (2004) used detailed data on North-South trade flows for pollution intensive product and provided the evidence for the hypothesis of pollution heaven [43]. As the world’s largest developing country, scholars have launched a fierce debate on whether China has become a pollution heaven for developed countries. Such as, Cai et al. (2018) found evidences that China has become a pollution heaven for 22 developed countries with the Belt and Road as a case study [44]. Shen et al. (2019) studied the relationship between the transfer of pollution-intensive industries and environmental efficiency in Guangdong province of China, and found that the migration of pollution-intensive was accompanied by pollution transfer, and the non-Pearl River Delta region has become a pollution haven for private investors in the Pearl River Delta [45]. Contrary to the pollution haven hypothesis, the pollution halo hypothesis argues that the technology spillover effect induced by the introduction of FDI can promote environmental technology innovation, which is beneficial to the improvement of environmental quality in the host country. Bartik (1988) selected US Fortune 500 companies from 1972 to 1978 as research objects and found that these companies are more concentrated in the areas with high environmental quality, partly indicating that the introduction of FDI is conducive to environmental protection [46]. Antweiler et al. (2001) found that the concentration of sulfur dioxide gradually decreased with the expansion of trade openness, indicating that trade is in favor of environmental quality improvement [47]. Jiang et al. (2017) conducted a spatial econometrical analysis on the cross-section data of 150 cities in China in 2014 and revealed that FDI has a significant positive technology spillover effect, which improves China’s air quality and supports the pollution halo hypothesis [48]. Wang (2019) based on 157 county-level data in Beijing-Tianjin-Hebei region in China and found that Beijing’s direct investment in its two neighboring provinces has a significant pollution halo effect, achieving a win-win situation for the regional economy growth and environmental protection [49]. Jiang et al. (2020) selected sulfur dioxide emission data of 270 prefecture-level cities in China from 2005 to 2016, and used spatial econometric models to investigate the socio-economic factors, and disclosed that FDI has a dramatical pollution halo effect, which is helpful to reducing sulfur dioxide pollution [50].

Given that the quality of the institution determines the quality of economic development to some extent, fiscal expenditure as an important institutional means of government macro-control is of great significance to high-quality economic development. Numerous studies have confirmed that there exist important linkages between fiscal expenditures and fiscal decentralization, local government coemption as well as “promotional incentives”, which might affect GTFP [51–53]. Taking different countries and regions as research objects, most scholars have reached a consistent conclusion that fiscal expenditure is not conducive to the growth of GTFP. For example, Bucovetsky (2005) believes that when fiscal expenditure is partial to infrastructure construction, despite that it could lead to the inflow of production factors, it also may stimulate a “zero-sum” competition between the local governments, and ultimately result in double losses in economic growth and environmental protection [54]. Glodsmith (2008) stated that the government nonproductive expenditure will have a crowing out effect on private investment [55]. According to the theory of multiplier, the economic growth rate may drop exponentially. However, some scholars disagreed with the above viewpoints, such as Megginsion and Netter (2001) considered that gradual institutional reforms are beneficial to the redistribution of resource elements and can improve environmental quality while promoting economic growth [56]. Since China’s tax-sharing reform in 1994, “promotional incentives” have simulated China’s local governments to use various fiscal policy tools (i.e. fiscal expenditure structure adjustment) to compete. This has led to the imbalance of China’s fiscal expenditure structure, which in turn resulted in factor market distortion and investment bias as well as environmental policies’ “race to the bottom” [57]. In this context, pollution emissions such as carbon emissions, sulfur dioxide and PM_2.5_ are inevitably rising rapidly, thereby creating a “green paradox” that is not conducive to China’s GTFP growth. Of course, some scholars holding different opinions, such as Song et al. (2020) argued that fiscal expenditure decentralization and competition could promote technological progress and thus contribute to China’s GTFP growth [58].

The aforementioned studies deeply explore the relationship between FDI, fiscal expenditure and GTFP, and provide insightful information for the growth of GTFP. However, such previous studies generally focused on a single issue, the impact of FDI on GTFP or the impact of fiscal expenditure on GTFP, ignoring the interaction between FDI and fiscal expenditure as well as its impact on GTFP. Moreover, the existing studies have not investigated the impact of FDI or fiscal expenditure on China’s GTFP from the perspective of spatial spillover, leading to their results are incomplete and some important information may be ignored. To fill these gaps, this paper attempts to incorporate FDI, fiscal expenditure and GTFP into a unified framework. Based on the theoretical mechanism analysis in the relationship between FDI and GTFP, fiscal expenditure and GTFP as well as the interaction between FDI and fiscal expenditure and GTFP, an empirical test is conducted with the panel data of China’s mainland 30 provinces (including autonomous regions and municipalities) from 2003 to 2017. This paper employs the spatial econometric model to evaluate the effects of FDI, fiscal expenditure, and their interaction items on the growth of China’s provincial GDP, on basis of which the spatial spillover effect and regional heterogeneity analysis are performed. A series of robustness tests and the endogeneity test can ensure the reliability of research results in this paper.

## 3. Theoretical analysis and research hypothesis

### 3.1 The impact of FDI on GTFP

FDI plays an impact on the host country’s GTFP through economic and environmental channels. In term of the economic channel, FDI has technological spillovers to the host country through competition effect, demonstration effect, industrial chain linkage effect and personal flow effect, which exert positive impacts on GTFP in the host country [59]. However, at the same time, the crowding-out effect of FDI on domestic enterprises could also inhibit GTFP improvement. Specifically, first, FDI introduction is generally accompanied by the spillover of green technology and advanced knowledge, which could intensify market competition in the host country, and the resulting competitive and catching-up effects will stimulate domestic enterprises to conduct technological innovations, improving the cleaning productivity of domestic enterprises, thereby promoting the host country’s GTFP [14]. However, FDI will also cause the foreign enterprises with more advanced technology and strong capital to quickly grab the host country’s domestic market share, thereby creating a crowding-out effect on the market space of the domestic enterprises. The strategy of market-for-technology is easy to form technological path dependence on foreign-funded enterprises, and domestic-funded enterprises lose their enthusiasm for innovation and output, which has a negative impact on GTFP. Second, foreign enterprises will achieve technological spillovers within and between industries through demonstration and industrial chain linkage effects, thereby promoting productivity and the host country’s GTFP. Third, foreign enterprises internalize advanced technology and management experience into the human capital value through professional training. Thus, the personnel flow between foreign and domestic enterprises is in favor of domestic enterprises’ technological innovation,and thereby improves the host country’s economic efficiency and GTFP.

With respect to the environmental channel, it is well known that China is closely linked to the global economy through foreign trade. However, while forming the “world-China” economic transfer process, the trade-induced environmental pollution is becoming more serious in China [47]. FDI affects environment mainly through scale effect and structural effect, which in turn affect the host country’s GTFP [60]. Specifically, first, FDI provides sufficient funds for the host country to expand production scale and obtain economic benefits, enabling the host country to carry out clean technology innovation, thereby promoting the host country’s technical efficiency and green technological progress, which is conducive to the improvement of the host country’s GTFP [61,62]. However, despite FDI could achieve scale economies effect, its huge energy consumption may also cause more pollution emissions, leading to the deterioration of the host country’s environmental quality and inhibiting the improvement of the host country’s GTFP. Second, FDI will have an impact on the host country’s industrial structure [63]. The more FDI is concentrated in high-polluting and low-tech industries,the higher the environmental costs it generates, yielding a pollution heaven effect. On the contrary, the more FDI is concentrated in low-polluting and high-tech industries, the more likely it is to produce spillover effect of clean technology. In the gathering area, a circular production system is formed, and promote regional industrial restructuring and improve environmental quality, form a "pollution halo".Thus, this will promote the improvement of the host country’s GTFP.

In addition, according to the “First Law of Geography”, FDI in a certain region not only affects the GTFP of the local region, but also affects the GTFP of its neighboring regions, that is the impact of FDI on GTFP has spatial spillover effect. To sum up, we propose the first hypothesis and its two competing sub-hypotheses as follows:

**Hypothesis 1:** FDI has dual impacts on GTFP and exists spatial spillover effect.
**Hypothesis 1a:** FDI promotes the improvement of GTFP in the local region and its neighboring regions by exerting technology spillover effects.
**Hypothesis 2a:** FDI restrains the development of GTFP in the local region and its neighboring regions by exerting scale and structural effects.

### 3.2 The impact of fiscal expenditure on GTFP

The impact of fiscal expenditure on GTFP is uncertain. On the one hand, government expenditures may provide a software and hardware development environment and financial support for technological innovation by issuing financial subsidies and increasing service expenditures, so as to achieve GTFP growth. First of all, by issuing financial subsidies to enterprises, local governments can solve the problem of excessively high technology research and development costs, promote overall industrial technological progress, and benefit the improvement of GTFP. Second, compared with the central government, local governments have more information advantages in local economic and social development, and can more effectively provide public goods that meet the preferences of local residents and adapt to local economic and social development. Local governments provide "hardware" conditions for promoting the promotion of regional GTFP by improving the efficiency of productive expenditures necessary for economic development such as public infrastructure. At the same time, local governments also expand consumption expenditures, service expenditures, and environmental governance expenditures through various forms of fiscal expenditures to maximize the interests of residents in their jurisdictions and promote high-quality economic development. On the other hand, large-scale fiscal expenditures may have negative externalities on GTFP. First of all, under the GDP-oriented performance evaluation system in China, the local officials may be “captured” by some interest groups [64,65], thereby the local government’s fiscal expenditures are too much concentrated on economic expenditures, ocusing on the expansion of economic quantity and neglecting the improvement of economic quality. The extensive economic development model leads to ecological deterioration, energy waste, and inhibits the growth of GTFP in the region. Second, fiscal expenditure will have a crowding-out effect on private investment. The profit margins of enterprises are compressed, leading to negative investment profits, which is not conducive to the green transformation of business methods, negatively affecting the technology investment structure, which inhibits the improvement of GTFP [49,50]. Third, the distortion of fiscal expenditure structure may induce market segmentation, which in turn causes resource misallocation and inefficiency in the spatial allocation of factors, such as limited spillover of knowledge and human capital, which plays a negative impact on labor productivity and economic growth.It also causes resource misallocation and increased energy consumption and pollution emissions, thereby generating significant negative environmental externalities, which hinders the growth of GTFP.

On the whole, fiscal expenditure has dual effects on GTFP, and the comprehensive impact is highly uncertain. In addition, under the decentralization system, there is a phenomenon of horizontal competition in the fiscal expenditure of various local governments, so the adjustment of the fiscal structure in the region can not only affect the development of GTFP in the region. At the same time, it can also influence the spatial allocation efficiency of neighboring local government behaviors, technology, human capital and other elements through learning and demonstration effects, thereby acting on the GTFP in spatially related areas. In light of this, we propose the second hypothesis and its two competing sub-hypotheses below:

**Hypothesis 2:** Fiscal expenditure has dual effects on GTFP and exists spatial spillover effect.
**Hypothesis 2a:** By providing more public goods services and financial support in line with local preferences, local governments optimize the allocation of resources and the new level of technology to promote the promotion of GTFP in the region. At the same time, the learning and demonstration effects produced will promote the growth of GTFP in spatially related areas.
**Hypothesis 2b:** Fiscal expenditure may promote extensive economic development, focusing solely on economic growth while ignoring energy conservation and environmental protection, hindering the growth of GTFP in the region, and at the same time leading to market segmentation between regions and increasing marginal costs, which will negatively affect GTFP growth in neighboring regions.

### 3.3 The interaction of FDI and fiscal expenditure and its impact on GTFP

With the inflow of FDI, the scale of local government fiscal expenditures has changed. At the same time, FDI has a screening effect on fiscal expenditures, and GTFP can be improved by improving the structure and efficiency of local expenditures. Specifically, on the one hand, according to Wagner’s law, the more FDI, the faster the national income will increase, thereby promoting the expansion of fiscal expenditure, increasing the proportion of environmental protection fiscal expenditure and the efficiency of fiscal expenditure, thereby promoting green development and GTFP promotion. On the other hand, FDI also puts forward higher requirements on the structure of fiscal expenditure.Local government will improve independent innovation capabilities and human capital levelby increasing the fiscal expenditures in R&D and education, so as to improve technological absorptive capacity maximize and thus enhance GTFP [66].

Although FDI inflows have great potential to promote the development of GTFP, the realization of this potential is in turn restricted by the local government’s fiscal spending preferences. On the one hand, under the pressure of political promotion, the local government’s expenditure structure is still more biased towards infrastructure investment rather than human capital and other soft environment construction investment (Zhang, 2010), which hinders the spillover effect of local FDI and affects the development of GTFP. On the other hand, some local government officials participate in intergovernmental competition by increasing fiscal expenditures. Excessive competition has produced a lot of sunk costs, compressed social expenditures, and caused a waste of resources. The resulting loss of efficiency may offset the growth effect of FDI on GTFP. At the same time, the shrinking social expenditure will also weaken the technology spillover effect of FDI in the region by affecting the development of science, education, culture and health industry, causing GTFP to suffer huge losses.

In addition, since both FDI and fiscal expenditure have significant spatial correlation characteristics, the interaction between the two inevitably produces spatial spillover effects between regions. At present, China’s regional ties are becoming increasingly close and interactions are becoming more frequent. While FDI and fiscal expenditures are synergistically affecting GTFP in the region, various innovations such as new knowledge, new inventions, and new technologies are overflowing. In addition, various local governments continuously adjust the structure of fiscal expenditures through competition, cooperation, and imitation, so as to have a comprehensive effect on economic growth, resource utilization and environmental protection in spatially related areas.To sum up, the third hypothesis and its two competing sub-hypotheses are proposed as follows:

**Hypothesis 3:** The interaction of FDI and fiscal expenditure has dual impacts on GTFP and exist spatial spillover effects.
**Hypothesis 3a:** The inflow of FDI can improve the fiscal expenditure structure of the region, increase the proportion of science and education expenditures in fiscal expenditures, provide good technical environmental conditions for the improvement of GTFP in the region, and generate learning and demonstration effects for spatially related regions, thereby promoting the improvement of GTFP in neighboring regions.
**Hypothesis 3b:** Under the incentive of promotion, the imbalance of local government fiscal expenditure structure will hinder the spatial spillover effect of FDI, which will adversely affect the growth of GTFP in this region and neighboring regions. However, due to the different levels of development between regions, this negative impact also shows certain differences in different regions.

## 4. Research design and data description

### 4.1 Baseline model specification

The new growth theory believes that technology is endogenous, and knowledge and human capital have spillover effect. Based on this, it is assumed that GTFP is not only affected by FDI but also depends on the average accumulation level of human capital. To this end, the production function can be constructed as follows:

Y=A(FDI,HR,t)×F(K,L) (1)

where *Y*, *FDI*, *HR*, *t*, *K* and *L* represents economic output, foreign direct investment, human capital, time, capital and labor input, respectively. *A*(.) is a standard Hicks-neutral efficiency that allows for exogenous shifts in the production function.

This paper follows the multivariate combination assumption proposed by Hulten et al. (2006) [67], which is shown as follows:

$$A(FDI,HR,t)=A_{i,0}e^{\lambda_{i}t}FDI_{i,t}^{\tau_{i}}\times HR_{i,t}^{\eta_{i}}$$ (2)

Substitute Eq (2) into Eq (1) to get Eq (3):

$$Y=A_{i,0}e^{\lambda_{i}t}FDI_{i,t}^{\tau_{i}}\times HR_{i,t}^{\eta_{i}}\times F(K_{it},L_{it})$$ (3)

where *i* and *t* denote region and time; the parameter *A*_*i*,0_ stands for initial production efficiency level; *λ*_*i*_ is the exogenous rate of productivity change, *τ*_*i*_ and *η*_*i*_ represent the impact parameters of FDI and human capital on GTFP, respectively.

According to the definition of GTFP, divide both ends of Eq (3) and get Eq (4) as follows:

$$GTFP_{i,t}=Y_{it}/F(K_{it},L_{it})=A_{i,0}e^{\lambda_{i}t}FDI_{i,t}^{\tau_{i}}\times HR_{i,t}^{\eta_{i}}$$ (4)

Take the natural logarithm form for both sides of Eq (4) and get Eq (5) bellow:

$$\ln GTFP_{i,t}=\ln Y_{it}/F(K_{it},L_{it})=\ln A_{i,0}+\lambda_{i}t+\tau_{i}\ln FDI_{i,t}+\eta_{i}\ln HR_{i,t}$$ (5)

where *GTFP* represents green total-factor productivity; ln*A*_*i*,0_ indicates the initial GTFP considering energy and environmental factors.

Add the interaction between FDI and fiscal expenditure into Eq (5) to get Eq (6) as follows:

$$\ln GTFP_{i,t}=\ln A_{i,0}+\lambda_{i}t+\tau_{i}\ln FDI_{i,t}+\omega_{i}FE_{i,t}+\delta_{i}\ln FDI_{i,t}\times FE_{i,t}+\eta_{i}\ln HR_{i,t}$$ (6)

where *FE* and *lnFDI×FE* stand for fiscal expenditure and the interaction between FDI and fiscal expenditure, respectively; *ω*_*i*_ and *δ*_*i*_ represents the coefficients of *FE* and ln*FDI*×*FE*, respectively.

Finally, the related control variables are introduced, and some of them are transformed into logarithmic form, and the benchmark model is built as shown in Eq (7):

$$\begin{array}{l} \ln GTFP_{i,t}=\alpha_{0}+\alpha_{1}\ln FDI_{i,t}+\alpha_{2}FE_{i,t}+\alpha_{3}\ln FDI_{i,t}\times FE_{i,t}+\beta_{1}\ln HR_{i,t}+\beta_{2}\ln ER_{i,t}\\ \;+\beta_{3}\ln PGDP_{i,t}+\beta_{4}\ln TD_{i,t}+\beta_{5}URBAN_{i,t}+\beta_{6}\ln TECH_{i,t} \end{array}$$ (7)

where ER, PGDP, TD, URBAN and TECH respectively denotes environmental regulation, GDP per capita, trade dependence, urbanization level and technological progress; *α*_*i*_(*i* = 0,1,2,3) and *β*_*j*_(*j* = 1,..,6) represent the coefficients of explanatory variables.

### 4.2 Spatial econometric model construction

Spatial econometric model mainly includes spatial Durbin model (SDM), spatial autoregressive model (SAM), spatial lag model (SLM) and spatial error model (SEM). Given that the SDM model includes spatial lag model and spatial error model in content, therefore it can effectively test the spatial spillover effect of the influencing factors of GTFP. Considering this, SDM is employed in this paper and constructed below:

$$\begin{array}{c} GTFP_{i,t}=\alpha_{0}+\rho W_{i,t}GTFP_{i,t}+\alpha_{1}\ln FDI_{i,t}+\alpha_{2}FE_{i,t}+\alpha_{3}\ln FDI_{i,t}\times FE_{i,t}+\beta_{1}\ln HR_{i,t}\\ +\beta_{2}\ln ER_{i,t}+\beta_{3}\ln PGDP_{i,t}+\beta_{4}\ln TD_{i,t}+\beta_{5}URBAN_{i,t}+\beta_{6}\ln TECH_{i,t}\\ +\theta_{1}W_{i,t}\ln FDI_{i,t}+\theta_{2}W_{i,t}FE_{i,t}+\theta_{3}W_{i,t}\ln FDI_{i,t}\times FE_{i,t}+\mu_{i}+\nu_{t}+\varepsilon_{it} \end{array}$$ (8)

where *W*_*i*,*t*_ is standardized spatial weight matrix; *ρ*_i_ denotes spatial autoregressive coefficient of GTFP, which represents the spatial impact of GTFP in the neighboring regions on the GTFP in region i; *α*_*i*_(*i* = 1,2,3) and *θ*_*j*_(*j* = 1,…,6) indicate the direct and indirect effects of each explanatory variable on the GTFP, respectively; *μ*_*i*_ represents the individual features that do not change with time; and *ν*_*t*_ denotes the time features that do not change with individual; *ε*_*it*_ stands for random error term that satisfies independent identical distribution and has finite variance.

### 4.3 Variables and data description

#### (1) Explained variable

In this paper, GTFP is considered as explained variable and GML productivity index derived from DDF is used to measure it for China’s mainland 30 provinces. Specifically, each province is deemed as decision making unit (DMU); capital stock, labor force and energy consumption are chosen as input variables, provincial gross domestic product (GDP) is selected as desirable output; undesirable outputs are represented by provincial emissions of sulfur dioxide (SO_2_) and chemical oxygen demand (COD).

According to the previous studies, this paper chooses capital stock as the proxy of capital input. Since the data of China’s provincial capital stock cannot be obtained directly, we utilize the perpetual inventory method to estimate them, and the formula is shown below:

$$K_{i,t}=K_{i,t-1}(1-\delta )+I_{i,t}$$ (9)

where *K*_*i*,*t*_ and *K*_*i*,*t*−1_ denote the capital stock of the *i*th province at time *t* and *t*-1. *I*_*i*,*t*_ stands for the total volume of the investment in fixed assets at time t, and *δ* is the depreciation rate. It should be noted that we use 2000 as the base year for the calculation of China’s provincial capital stock, and the depreciation rate is set at 9.5%.

The data of China’s provincial labor force and GDP are collected from China’s Statistical Yearbook (2004–2018), and the data on energy consumption are obtained from China’s Energy Statistical Yearbook (2004–2018) and units of all sorts of primary energy (such as coal, oil, gas and electricity) are converted into tons of standard coal equivalent (TSCE). The data on SO_2_ and COD emissions are directly collected from China Statistical Yearbook (2004–2018) and China Environmental Statistical Yearbook (2004–2018).

It is constructed that the production possible set *P*^*t*^(*x*^*t*^) based on the data of the *t*th (*t* = 1, 2, …, *T*) period of China’s mainland provinces, which can be written as follows:

$$P^{t}\left(x^{t}\right)=\{\left(y^{t},b^{t}\right):x^{t}can\;produce\;(y^{t},b^{t})\}$$ (10)

Then, the global production technology set *P*^*G*^(*x*) can be constructed as follows:

$$P^{G}\left(x\right)=P^{1}\left(x^{1}\right)\cup P^{2}\left(x^{2}\right)\cup ,\cdots ,\cup P^{T}\left(x^{T}\right)$$ (11)

According to Oh (2010) [68], GML productivity index used to measure GTFP can be defined as follows:

$$GML_{k}^{t,t+1}(x^{t},y^{t},b^{t};x^{t+1},y^{t+1},b^{t+1})=\frac{1+D^{G}(x^{t},y^{t},b^{t};g)}{1+D^{G}(x^{t+1},y^{t+1},b^{t+1};g)}$$ (12)

where k denotes the province to be evaluated; *D*^*G*^(•) stands for global DDF and it can be calculated by DEA approach. Given that we aim to the maximization of desirable output while minimizing inputs and undesirable outputs, the direction vector *g* is set as (−*x*,*y*,−*b*). If the GML index is greater than 1, equal to 1 and less than 1, it means that the GTFP of the province to be evaluated improves, remains unchanged, and decreases from *t* to *t*+1.

In order to intuitively show the differences and spatial correlation characteristics of China’s provincial GTFP, this paper selects the GTFP data of three typical years in 2004, 2010 and 2017 provided in Table 1. it can be seen that China’s GTFP has significant regional distribution heterogeneity.

**Table 1:** China’s provincial GTFP in 2004, 2010 and 2017.

Provinces	GTFP2004	GTFP2010	GTFP2017
Heilongjiang	1.1197	1.30372	1.06966
Xinjiang	1.10419	1.70232	1.40217
Shanxi	1.15022	1.29649	1.24349
Ningxia	1.11659	1.65114	1.11363
Shandong	1.15266	1.48445	1.608
Henan	1.17094	1.23782	1.12879
Jiangsu	1.08508	1.61915	1.90626
Anhui	1.12992	1.47768	1.58457
Hubei	1.13625	1.76654	1.86688
Zhejiang	1.08499	1.50895	1.69702
Jiangxi	1.10982	1.40599	1.58124
Hunan	1.14581	1.54618	1.62793
Yunnan	1.13719	1.43559	1.1813
Guizhou	1.10998	1.8834	1.97659
Fujian	1.04462	1.30925	1.35535
Guangxi	1.13916	1.16301	1.05662
Guangdong	1.11857	1.47986	1.51849
Hainan	1.0907	1.55239	1.42647
Jilin	1.08794	1.03091	0.906691
Liaoning	1.01655	1.22285	0.953053
Tianjin	1.13296	1.55677	1.88224
Qinghai	1.10572	1.55823	1.28739
Gansu	1.12927	1.53047	1.4025
Shanxi	1.16169	1.65389	1.74296
Inner Mongolia	1.05556	1.20046	0.812103
Chongqing	1.08455	1.41494	1.72571
Hebei	1.15773	1.40298	1.2924
Shanghai	1.13712	1.63604	1.91735
Beijing	1.12368	1.73282	1.92148
Sichuan	1.13258	1.60598	1.85751

#### (2) Core explanatory variables

Foreign direct investment (FDI) and fiscal expenditure (FE) are considered as two core explanatory variables in this paper. Regarding FDI, following most previous studies, this paper adopts the proportion of total foreign direct investment to provincial GDP to measure FDI, and Table 2 illustrates the geographic distribution features of China’s provincial FDI in 2010 and 2017. With regard to FE, we take the ratio of local per capita fiscal expenditure to the national per capital fiscal expenditure as its proxy variable, and the geographic distribution maps of China in 2010 and 2017 are displayed in Table 3. It can be found the spital distributions of FDI and FE vary greatly across different provinces in China.

**Table 2:** China’s provincial FDI in 2004, 2010 and 2017.

Provinces	FDI2004	FDI2010	FDI2017
Heilongjiang	1.1197	1.30372	1.06966
Xinjiang	1.10419	1.70232	1.40217
Shanxi	1.15022	1.29649	1.24349
Ningxia	1.11659	1.65114	1.11363
Shandong	1.15266	1.48445	1.608
Henan	1.17094	1.23782	1.12879
Jiangsu	1.08508	1.61915	1.90626
Anhui	1.12992	1.47768	1.58457
Hubei	1.13625	1.76654	1.86688
Zhejiang	1.08499	1.50895	1.69702
Jiangxi	1.10982	1.40599	1.58124
Hunan	1.14581	1.54618	1.62793
Yunnan	1.13719	1.43559	1.1813
Guizhou	1.10998	1.8834	1.97659
Fujian	1.04462	1.30925	1.35535
Guangxi	1.13916	1.16301	1.05662
Guangdong	1.11857	1.47986	1.51849
Hainan	1.0907	1.55239	1.42647
Jilin	1.08794	1.03091	0.906691
Liaoning	1.01655	1.22285	0.953053
Tianjin	1.13296	1.55677	1.88224
Qinghai	1.10572	1.55823	1.28739
Gansu	1.12927	1.53047	1.4025
Shanxi	1.16169	1.65389	1.74296
Inner Mongolia	1.05556	1.20046	0.812103
Chongqing	1.08455	1.41494	1.72571
Hebei	1.15773	1.40298	1.2924
Shanghai	1.13712	1.63604	1.91735
Beijing	1.12368	1.73282	1.92148
Sichuan	1.13258	1.60598	1.85751

**Table 3:** China’s provincial FE in 2004, 2010 and 2017.

Provinces	FE2004	FE2010	FE2017
Heilongjiang	1.1197	1.30372	1.06966
Xinjiang	1.10419	1.70232	1.40217
Shanxi	1.15022	1.29649	1.24349
Ningxia	1.11659	1.65114	1.11363
Shandong	1.15266	1.48445	1.608
Henan	1.17094	1.23782	1.12879
Jiangsu	1.08508	1.61915	1.90626
Anhui	1.12992	1.47768	1.58457
Hubei	1.13625	1.76654	1.86688
Zhejiang	1.08499	1.50895	1.69702
Jiangxi	1.10982	1.40599	1.58124
Hunan	1.14581	1.54618	1.62793
Yunnan	1.13719	1.43559	1.1813
Guizhou	1.10998	1.8834	1.97659
Fujian	1.04462	1.30925	1.35535
Guangxi	1.13916	1.16301	1.05662
Guangdong	1.11857	1.47986	1.51849
Hainan	1.0907	1.55239	1.42647
Jilin	1.08794	1.03091	0.906691
Liaoning	1.01655	1.22285	0.953053
Tianjin	1.13296	1.55677	1.88224
Qinghai	1.10572	1.55823	1.28739
Gansu	1.12927	1.53047	1.4025
Shanxi	1.16169	1.65389	1.74296
Inner Mongolia	1.05556	1.20046	0.812103
Chongqing	1.08455	1.41494	1.72571
Hebei	1.15773	1.40298	1.2924
Shanghai	1.13712	1.63604	1.91735
Beijing	1.12368	1.73282	1.92148
Sichuan	1.13258	1.60598	1.85751

#### (3) Control variables

To improve the accuracy of regression models, the following control variables are introduced in this paper.

1. Human capital level (HR). As a carrier of knowledge and skills, human capital plays an important impact on GTFP [69]. In addition, the spillover effects of FDI and fiscal expenditure also depend on the level of human capital. Therefore, following Barro and Lee (1993), provincial average education level of residents is utilized to measure HR [70].
2. Environmental regulation (ER). We introduce ER to test the existence of the Porter hypothesis, that is, whether environmental regulation can improve GTFP in China, and the proportion of industrial pollution control investment to provincial GDP is chosen to measure ER [71].
3. Economic development level (PGDP). The impacts of FDI and fiscal expenditure on GTFP are both affected by the level of provincial economic development. Given that the actual GDP per capita can well reflect the scale of regional scale and residents’ living standards, the per capita GDP at 2000 constant price is selected to reflect PGDP.
4. Trade openness (TD). Trade openness affects provincial GTFP by influencing the scale and speed of FDI introduction as well as its technological spillover effect, and the ratio of provincial total import and export to GDP is used to measure TD [62].
5. Urbanization (URBAN). Because urbanization will bring population and industrial agglomeration, thereby leading to an increase in energy consumption and pollution emissions, and thus play a negative effect on GTFP [66,72]. The proportion of provincial urban population in total population is chosen to measure URBAN.
6. Technological progress (TECH). Technological progress can directly improve production efficiency, which can promote GTFP improvement [73,74] Given that the most closely related to technological innovation is the patent granted for invention, thus we use provincial number of patents granted for invention as a proxy variable to measure TECH [75].

All the related data on above variables are collected from China Statistical Yearbook (2001–2018), China Regional Economic Statistical Yearbook (2004–2018) and China Science and Technology Statistical Yearbook (2004–2018). To reduce heteroscedasticity, some variables are converted into the logarithmic forms. The descriptive statistics of the above variables are shown in Table 4.

**Table 4:** Descriptive statistics of the explained and explanatory variables.

Variables	Average	Std.error	Minimize	Maximize
*GTFP*	1.382	0.261	0.812	1.977
*lnFDI*	-1.310	0.869	-3.051	1.768
*FE*	1.202	0.631	0.556	4.756
*LnFDI×FE*	-1.356	1.204	-5.984	2.881
*lnHR*	2.151	0.115	1.798	2.526
*lnER*	0.160	0.536	-2.996	1.539
*lnPGDP*	10.23	0.719	8.212	11.77
*lnTD*	-1.691	0.987	-4.075	0.543
*URBAN*	0.517	0.144	0.248	0.896
*lnTECH*	2.249	0.180	1.573	2.592

### 4.4 Spatial weight matrix construction

The selection of the spatial weight matrix is of great significance to Moran’s I spatial correlation test and spatial econometric analysis. In order to ensure the robustness of spatial econometric model results, we will use different spatial weight matrices for spatial econometric regression. Given that under the background of competition between the local governments, both the technological spillover effect from FDI and fiscal expenditure have significant characteristics of spilling over to the neighboring regions, so the spatial spillover effect could be investigated by the geographical adjacency matrix. In addition, considering the economic feature of GTFP, the economic distance matrix with considering geographical factors could also be used to analyze the spatial spillover effects. Based on the above analysis, four kinds of spital weight matrix, namely geographic adjacency matrix (*W*_1_), economic distance matrix (*W*_2_), geographic distance matrix (*W*_3_) as well as geographic and economic distance nested matrix (*W*_4_) are introduced to the spatial econometric analysis in this paper, and they are defined as follows:

$$W_{1}=\left\{ \begin{array}{l} 1,\;region\;i\;is\;adjacent\;to\;region\;j\\ 0,\;region\;i\;is\;not\;adjacent\;to\;region\;j \end{array}\right.$$ (13)

$$W_{2}=\left\{ \begin{array}{c} \frac{1}{\left|x_{i}‐x_{j}\right|+1},\;i\neq j\\ \;1,\;i=j \end{array}\right.$$ (14)

$$W_{3}=\left\{ \begin{array}{l} \frac{1}{d^{2}},\;i\neq j\\ 1,\;i=j \end{array}\right.$$ (15)

$$W_{4}=W_{2}\times W_{3}$$ (16)

where *i* and *j* stand for different regions; *d* represents the distance between the geographic centers of the two regions; *x* denotes regional GDP per capita. *W*_1_ is a 0–1 matrix constructed based on whether the two regions are adjacent or not; *W*_2_ is an economic distance matrix constructed based on the per capita GDP of the two regions; *W*_3_ is a geographic distance matrix constructed based on the distance between the geographic centers of the two regions; *W*_4_ is obtained by multiplying *W*_2_ and *W*_3_ matrix, and the matrices used for spatial econometric analysis in this paper have been standardized.

## 5. Results and discussion

### 5.1 Global and local spatial autocorrelation analysis

Before the spatial econometric analysis, it is necessary to test the spatial dependence and correlation of related variables. Thus, we apply the widely used global Moran’s index (Moran’s I) to test the spatial autocorrelation for related variables, and its formula is provided as follows:

$$Moran's\;I=\frac{\sum_{i=1}^{n}\sum_{j=1}^{n}W_{ij}(\ln Y_{i}-\ln\bar{Y})(\ln Y_{j}-\ln\bar{Y})}{S^{2}\sum_{i=1}^{n}\sum_{j=1}^{n}W_{ij}}$$ (17)

where $S^{2}=\sum_{i=1}^{n}{\left(\ln Y_{i}-\ln\bar{Y}\right)}^{2}/n$; *Y* is the variable to be analyzed and $\bar{Y}$ is all sample average; *n* is the number of provinces; *W*_*ij*_ is geographic adjacency matrix. The Moran’s I index is between -1 and 1. If it ranges from -1 to 0, it indicates that there is a negative spatial correlation for the variable to be analyzed, manifesting as a high-low or low-high cluster in the second and fourth quadrants; if it is between 0 and 1, it means positive spatial correlation exist, which is manifested as a high-high or low-low cluster of the first or third quadrants; if it equals to 0, it means that there is no spatial autocorrelation. Table 5 shows the spatial correlation features of *GTFP*, *lnFDI*, *FE* and *lnFDI×FE* in China from 2003 to 2017.

**Table 5:** The Moran’s I indexes of *GTFP*, *lnFDI*, *FE* and lnFDI×FE.

period	*GTFP*	*lnFDI*	*FE*	*lnFDI*×*FE*
2003	0.145*	0.342***	0.166*	0.304***
	(0.067)	(0.002)	(0.054)	(0.003)
2004	0.211**	0.386***	0.170**	0.347***
	(0.039)	(0.001)	(0.047)	(0.001)
2005	0.138	0.435***	0.167*	0.396***
	(0.158)	(0.000)	(0.051)	(0.000)
2006	0.162	0.398***	0.183**	0.397***
	(0.105)	(0.000)	(0.042)	(0.000)
2007	0.290***	0.317***	0.182**	0.380***
	(0.007)	(0.003)	(0.045)	(0.000)
2008	0.302***	0.313***	0.197**	0.425***
	(0.005)	(0.003)	(0.036)	(0.000)
2009	0.320***	0.322***	0.216**	0.441***
	(0.004)	(0.003)	(0.028)	(0.000)
2010	0.277***	0.296***	0.208**	0.432***
	(0.010)	(0.003)	(0.039)	(0.000)
2011	0.305***	0.409***	0.188*	0.562***
	(0.005)	(0.000)	(0.062)	(0.000)
2012	0.318***	0.421***	0.181*	0.572***
	(0.004)	(0.000)	(0.070)	(0.000)
2013	0.353***	0.470***	0.201**	0.526***
	(0.002)	(0.000)	(0.048)	(0.000)
2014	0.384***	0.466***	0.195*	0.515***
	(0.001)	(0.000)	(0.054)	(0.000)
2015	0.428***	0.437***	0.183*	0.481***
	(0.000)	(0.000)	(0.065)	(0.000)
2016	0.448***	0.376***	0.197**	0.466***
	(0.000)	(0.001)	(0.049)	(0.000)
2017	0.452***	0.388***	0.154	0.451***
	(0.000)	(0.001)	(0.103)	(0.000)

Note: p-values are in parentheses

***, **, and * represent significant level of 1%, 5% and 10%, respectively.

As shown in Table 5, the global Moran’s I indexes of *GTFP*, *lnFDI*, *FE* and *lnFDI×FE* in China are all greater than 0 from 2003 to 2017, and all of them have passed the 5% level significance test in most years. This indicates that the spatial distribution of these variables is non-random, and confirms the existence of spatial positive autocorrelation, showing that it is necessary to utilize spatial econometric model for the exploration of the impact of FDI and FE on China’s provincial GTFP.

**Fig 1:**
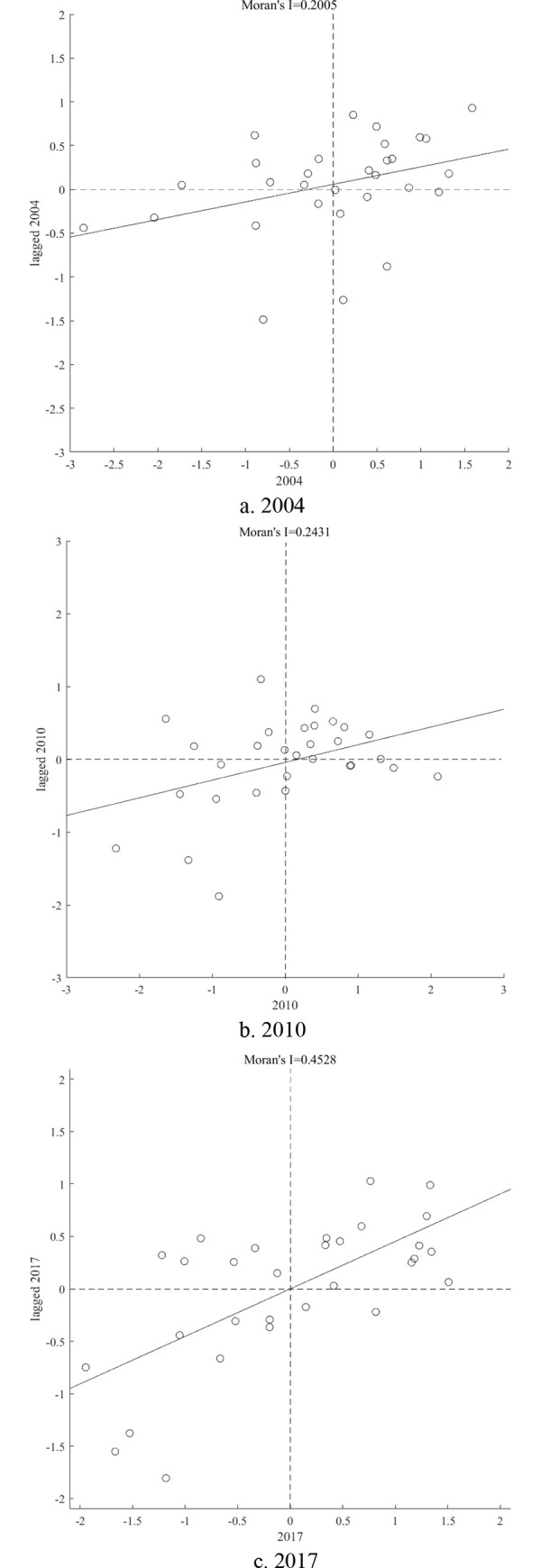
Local Moran scatter plots of China’s provincial GTFP in 2004, 2010 and 2017.

In order to further explore the spatial correlation feature of China’s provincial GTFP, on the basis of global spatial autocorrelation analysis, we apply the local Moran scatter plots to analyze the spatial correlation effects of China’s provincial GTFP with 2004, 2010 and 2017 as the research years, and the results are shown in Fig 1.

It can be found in Fig 1 that in 2004, China’s 17 provinces are in the first and third quadrants, indicating that their GTFPs have a high degree of positive spatial correlation. Among them, 12 provinces are located in the first quadrant and displays the feature of high GTFP and high spatial lag (high-high, HH), and 5 provinces located in the third quadrant and exhibit the feature of low GTFP and low spatial lag (low-low, LL). The remaining 13 provinces are located in the second and fourth quadrants, which indicates that their GTFPs negatively spatial correlated. Among them, 6 provinces in the second quadrant, showing the feature of high GTFP and low spatial lag (high-low, HL), and 7 provinces in the fourth quadrant exhibit the feature of low GTFP and high spatial lag (low-high, LH). Similarly, it can be found that in 2010, the GTFP of China’s 18 provinces show positive spatial correlation, the remaining 12 provinces exhibiting negative spatial correlation; in 2017, the GTFP of China’s 22 provinces are positively spatial correlated, and the remaining 8 provinces show negative spatial correlation.

From Table 6 and Fig 1, it is revealed that China’s provincial GTFP exhibits significant positive spatial correlation feature, indicating that provinces with higher (lower) GTFPs and their neighboring provinces also show higher (lower) GTFPs, displaying the spatial agglomeration feature of HH (LL). With the time passing, the HH and LL agglomeration features have become more and more obvious. Moreover, it also can be found that the provinces with HH agglomeration feature are mainly concentrated in the eastern economically developed region such as Shanghai, Jiangsu, etc; the provinces with LL agglomeration feature are mainly concentrated in the central and western regions such as Sichuan, Guangxi, Yunnan and Gansu, etc. On the whole, it is disclosed that China’s provincial GTFP exhibits the features of stable positive spatial spillover and significant regional heterogeneity.

**Table 6:** The spatial types of China’s provincial GTFP in 2004, 2010 and 2017.

Types	Provinces		
	2004	2010	2017
**HH**	Heilongjiang, Xinjiang, Henan, Anhui, Guizhou, Fujian, Hainan, Liaoning, Qinghai, Inner Mongolia, Hebei, Shanghai	Xinjiang, Ningxia, Jiangsu, Anhui, Hunan, Hainan, Jilin, Tianjin, Qinghai, Chongqing, Sichuan	Heilongjiang, Xinjiang, Ningxia, Jiangsu, Anhui, Zhejiang, Jiangxi, Hunan, Jilin, Qinghai, Shanxi, Inner Mongolia, Shanghai
**HL**	Shanxi, Ningxia, Hubei, Gansu, Beijing, Sichuan	Shanxi, Hubei, Jiangxi, Guizhou, Inner Mongolia, Shanghai	Hubei, Guizhou
**LH**	Jiangxi, Hunan, Yunnan, Jilin, Tianjin, Shanxi, Chongqing	Heilongjiang, Henan, Zhejiang, Fujian, Liaoning, Shanxi	Shandong, Henan, Hunan, Fujian, Liaoning, Chongqing
**LL**	Shandong, Jiangsu, Zhejiang, Guangxi, Guangdong	Shandong, Yunnan, Guangxi, Guangdong, Gansu, Hebei, Beijing	Shanxi, Yunnan, Guangxi, Guangdong, Tianjin, Gansu, Hebei, Beijing, Sichuan

### 5.2 Baseline spatial econometric regression results

Based on theoretical and spatial autocorrelation analysis above, this paper further empirically explores the relationship between FDI, FE and GTFP through spatial econometric model using the panel data of China’s mainland 30 provinces from 2003 to 2017 in this section. Through the Wald and LM tests, it shows that SDM model is more appropriate in this paper. Furthermore, the result of Hausman test ensures that fixed-effect model is a better choice. Therefore, the fixed-effect SDM is employed as the baseline model for empirical analysis in this paper, and Table 7 reports the regression results.

**Table 7:** The results of baseline SDM, SAR and SEM regression.

Variables	SDM	SAR	SEM
*lnFDI*	0.109***	0.124***	0.046
	(0.031)	(0.030)	(0.031)
*FE*	-0.101***	-0.071***	-0.111***
	(0.023)	(0.021)	(0.022)
*lnFDI×FE*	-0.028	-0.033*	0.023
	(0.020)	(0.020)	(0.020)
*W×lnFDI*	0.447***		
	(0.069)		
*W×FE*	-0.140**		
	(0.057)		
*W×(LnFDI×FE)*	-0.259***		
	(0.045)		
*lnHR*	0.407**	0.359*	0.398*
	(0.186)	(0.184)	(0.205)
*lnER*	-0.047***	-0.046***	-0.040***
	(0.013)	(0.013)	(0.012)
*lnPGDP*	0.342***	0.278***	0.436***
	(0.038)	(0.037)	(0.049)
*lnTD*	0.041**	0.024	-0.022
	(0.020)	(0.020)	(0.021)
*URBAN*	-1.857***	-1.858***	-1.808***
	(0.298)	(0.298)	(0.299)
*lnTECH*	-0.020	-0.006	-0.005
	(0.019)	(0.018)	(0.020)
*ρ or λ*	0.551***	0.643***	0.720***
	(0.042)	(0.038)	(0.036)
*Sigma*^*2*^	0.009***	0.010***	0.009***
	(0.001)	(0.001)	(0.001)
*R*^*2*^	0.034	0.200	0.150
*Hausman*	47.34***	37.11***	13.19
	(0.000)	(0.000)	(0.213)
*Wald Test*	43.66***		
	*(0*.*000)*		
*LR Test*	57.87***		
	*(0*.*000)*		

**Note:** Robust standard deviations are in parentheses

***, **, and * represent significance level of 1%, 5% and 10%, respectively.

From Table 7, it can be seen that the spatial autocorrelation coefficient ρ is 0.534, and significant at the 1% significance level, indicating that China’s provincial GTFP has a feature of significant positive spatial correlation, which is consistent with the result of Moran’s I index above. According to column 2 and 3 in Table 7, it can be preliminarily judged that the introduction of FDI promotes the improvement of GTFP in the local and its neighboring provinces; fiscal expenditure inhibits the local and its neighboring provinces GTFP improvement; the interaction of FDI and fiscal expenditure also inhibits the improvement of GTFP in the local and its neighboring provinces. In addition, most control variables have passed the significance test, indicating that the explanatory variables selected in the baseline regression model are reasonable and can effectively avoid the bias of omitted variables

It is well known that when there is a spatial spillover effect, the change of a certain influencing factor will not only cause changes the local GTFP, but also affect the GTFP of its neighboring regions. Therefore, with respect to Lesage and Pace (2009), the effect of each factor on the GTFP can be further decomposed into direct and indirect effects [76]. The 3th and 4th columns of Table 8 respectively report the estimated results of direct and indirect effects.

**Table 8:** The direct and indirect effects of SDM regression.

Variables	LR_Direct	LR_Indirect	LR_Total
*lnFDI*	0.199***	1.008***	1.207***
	(0.032)	(0.136)	(0.148)
*FE*	-0.136***	-0.391***	-0.528***
	(0.023)	(0.109)	(0.118)
*lnFDI×FE*	-0.075***	-0.541***	-0.616***
	(0.022)	(0.091)	(0.103)
*Control*	YES	YES	YES

**Note:** LR_Direct, LR_Indirect and LR_Total are the long-run direct effect indirect effect and total effect of the variables; Robust standard deviations are in parentheses

***, **, and * represent significance level of 1%, 5% and 10%, respectively. All these symbols are the same for the following tables.

The direct effect is defined as the impact of the factor on the local GTFP, and the indirect effect is defined as the impact of the factor on the neighboring GTFP, that is, spatial spillover effect. As shown in Table 8, it is revealed that the coefficients of direct effect, indirect effect and total effect of FDI are all significantly positive at the 1% significance level, and the indirect effect coefficient is 1.008, which is greater than the direct effect coefficient 0.199. This indicates that FDI introduction not only promotes the local GTFP, but also plays significantly positive spatial spillover effects on its neighboring regions, and the hypothesis 1a has been verified. It can be found that FDI introduction can significantly boost the local region GTFP. This is mainly because the inflow of FDI directly brings advanced management experience and green technology to the region, and quickly realizes the improvement of internal production efficiency and environmental pollution in the region through the effects of green technology spillovers, learning and demonstration, industrial chain linkage and personnel flow, thereby promoting the improvement of the local region GTFP [40]. At the same time, the inflow of FDI in this region has produced a greater spatial spillover effect on neighboring regions, indicating that in order to develop the economy of the jurisdiction, and they are racing to introduce preferential policies to participate in the competition of attracting investment, enhance their own technology absorption capacity, and significantly exert the positive externality of FDI spatial agglomeration, thereby effectively driving the improvement of GTFP in neighboring areas.

The coefficient of the direct and indirect effects of fiscal expenditure is significantly negative at the 1% significance level. This indicates that fiscal expenditure not only hinders the improvement of local GTFP, but also exerts a negative impact on its neighboring GTFP growth, and the hypothesis 2a has been verified. To pursue GDP growth and achieve personal promotion, local officials tend to invest more fiscal expenditures in the field of production, and by means of environmental “race to the bottom” to grab resources from their neighboring regions, causing resources misallocation and the decline of economic efficiency and environmental quality and thus hindering the local region GTFP growth. At the same time, the local government’s economic development model at the expense of the environment has produced a regional "demonstration" role, stimulating the fiscal expenditure structure of neighboring regions and tilting production fields, triggering vicious competition between regions. Therefore, the obstructive effect of fiscal expenditure on GTFP in neighboring areas will have a "spatial spillover" effect.

The coefficient of the direct and indirect effects of the interaction term between *lnFDI* and *FE* is significantly negative at 1% significance level, which indicates that the interaction between FDI and fiscal expenditure plays significantly negative effect on the local region and its neighboring regions GTFP. This may be because that the negative externality of fiscal expenditure is greater than the positive externality of FDI. The resources misallocation caused by the imbalance of the local region fiscal expenditure structure makes FDI unable to play the technological spillover effect normally, and thus leads to the decline of GTFP, and the hypothesis 3b has been verified. Despite that the direct effect of the interaction term is negative, it is smaller than that of fiscal expenditure. It shows that FDI’s pursuit of marketization will weaken the role of the local government in economic development, harden the government’s fiscal revenue and expenditure, help expand private investment, activate corporate vitality, and drive technological innovation and industrial upgrading in the region.Therefore FDI still plays a positive spatial spillover effect, which weakens the negative effect of fiscal expenditure on the local GTFP. However, the indirect effect of the interaction term is greater than that of fiscal expenditure. The reason for this phenomenon may be that neighboring regions have taken measures to relax environmental regulations to participate in FDI competition with limited local resource endowments. However, the imported FDI was concentrated in pollution-intensive industries, which increased the pressure of regional environmental pollution, and the loss of efficiency and increased pollution hindered the growth of regional GTFP.

With regard to control variables, the coefficients of *lnHR*, *lnPGDP* and *lnTD* are significantly positive at the 5% significance level, indicating these factors can effectively promote China’s GTFP growth. However, the coefficient of *lnER* is significantly negative at 1% significance level, which suggests that environmental regulation is not beneficial to GTFP growth, the Porter hypothesis is not supported here; the coefficient of *URBAN* is significantly negative at 1% significance level, indicating that urbanization quality is relatively low in China, and the urbanization process is accompanied by a large amount of energy consumption and pollution emissions, which inhibits GTFP growth; the coefficient of *lnTECH* is negative but not significant at 10% significance level, which is not consistent with our expectation. Given the bias of technological progress largely determines the influencing direction of technological progress on GTFP, the regression result obtained in this paper indicates that China’s R&D investment may be used more to promote production technology rather than green technology, leading to the expansion of production scale and thus hindering the growth of GTFP.

### 5.3 Regional heterogeneity analysis

To investigate the regional heterogeneity in the impact of FDI, fiscal expenditure on GTFP in China, we divide China’s mainland 30 provinces into two major regions: The Eastern and the Central-Western regions, to perform spatial econometric regression analysis. The Eastern region includes Beijing, Tianjin, Liaoning, Hebei, Shandong, Jiangsu, Shanghai, Zhejiang, Fujian, Guangdong and Hainan. The Central-Western region includes Heilongjiang, Jilin, Inner Mongolia, Shanxi, Henan, Anhui, Hubei, Hunan, Jiangxi, Guangxi, Guizhou, Yunnan, Sichuan, Chongqing, Shaanxi, Ningxia, Qinghai, Xinjiang and Gansu. Table 9 reports the regression results.

**Table 9:** The regression results of the Eastern region and the Central-Western region.

Variables	*the Eastern region*		*the Central-Western region*	
	*Coef*.	*S*.*D*.	*Coef*.	*S*.*D*.
*lnFDI*	0.077	0.048	0.091	0.075
*FE*	-0.149***	0.025	0.360**	0.144
*lnFDI×FE*	-0.020	0.030	0.016	0.055
*W×lnFDI*	0.517***	0.102	-0.434***	0.150
*W×FE*	-0.093*	0.053	1.410***	0.279
*W×(LnFDI×FE)*	-0.332***	0.066	0.437***	0.115
***LR_Direct***				
*lnFDI*	0.147***	0.048	0.072	0.077
*FE*	-0.166***	0.025	0.429***	0.144
*lnFDI×FE*	-0.062*	0.033	0.038	0.057
***LR_Indirect***				
*lnFDI*	0.629***	0.136	-0.457***	0.162
*FE*	-0.157***	0.052	1.643***	0.269
*lnFDI×FE*	-0.389***	0.087	0.490***	0.124
***LR_Total***				
*lnFDI*	0.777***	0.152	-0.385**	0.181
*FE*	-0.323***	0.065	2.072***	0.290
*lnFDI×FE*	-0.451***	0.105	0.529***	0.138
***ρ***	0.290***	0.070	0.195***	0.069
***Sigma2***	0.005***	0.001	0.012***	0.001
***R***^***2***^	0.097		0.000	

**Note:** Coef. is the coefficient of the explanatory variable; S.D. is the Robust standard deviation. All these symbols are the same for the following tables.

As shown in Table 9, the spatial autocorrelation coefficients of the east and the central-west are respectively 0.290 and 0.195, and they have passed the significant test under the 1% significance level, showing that there exists a dramatically spatial spillover effect in the two major regions. In addition, based on the regression results, it also can be found that the impact of FDI, fiscal expenditure and their interaction on GTFP exhibits obvious regional heterogeneity.

Specifically, first, the direct and indirect effects of FDI on the Eastern region are both significantly positive, the direct effect on the Central-Western region are positive but not significant, and the indirect effect are significantly negative. This shows that FDI not only can significantly promote the Eastern GTFP growth and but also can generate spatial spillovers to its neighboring regions. However, the effect of FDI on Central-Western GTFP is not significant, and it may even hinder its neighboring GTFP growth. The main reason may be that compared with the Central-Western region, the Eastern region is located on the coast and it is easier to attract FDI, which promotes local and its neighboring GTFP growth through learning effects. In contrast, the Central-Western region is located inland and it is more difficult to attract FDI [77]. To pursue economic growth, the Central-Western local governments tend to launch the competition of “race to the bottom”, leading to a large number of entries of FDI with high energy consumption and pollution emissions, which is not conducive to GTFP growth.

Second, the direct and indirect effects of fiscal expenditure on the Eastern GTFP are significantly negative, while they are both significantly positive in the Central-Western region. This indicates that the Eastern region has not abandoned the GDP-oriented growth mode currently, and its fiscal expenditure is still mainly invested in the production sector, and its proportion invested in environmental protection is relatively small, leading to the environmental deterioration effect is greater than the economic growth effect generated by the fiscal expenditure. Moreover, under the background of local governments competition, spatial spillover effects have been yielded, which hinders the local and its neighboring GTFP growth. However, similar to the Eastern region, the Central-Western fiscal expenditure is also mainly invested in production sector, but the economic growth effect is obviously greater than the environmental degradation effect generated by the fiscal expenditure due to the region’s weaker economic foundation. Therefore, on the whole, fiscal expenditure in the Central-Western region can promote the growth of regional GTFP.

Third, with regard to the impact of the interaction between FDI and fiscal expenditure on GTFP, it can be found that both direct and indirect effects in the Eastern region are significantly negative, while both of them are positive in the Central-Western region, but the direct effect is not significant. Additionally, the coefficient of lnFDI*×*FE is between the coefficient of lnFDI and the coefficient of FE, which shows that there is indeed an interaction between FDI and fiscal expenditure. In detail, the fiscal expenditure distortion in the Eastern region has weaken the positive effect of FDI on regional GTFP growth, and FDI has also partly reduced the negative effect of fiscal expenditure distortion on regional GTFP growth, while the interaction between them still plays a negative impact on regional GTFP. This is because fiscal expenditure distortion leads to resource misallocation, and its negative effect on regional GTFP offsets the positive effect of FDI on regional GTFP [78]. Compared with the Eastern region, the Central-Western region is more supported by the central fiscal expenditure, which stimulates the positive effect of FDI on regional GTFP, making FDI and fiscal expenditure synergistically promote regional GTFP growth. However, due to its weaker economic foundation, this synergy has not been fully realized.

### 5.4 Robustness test

To ensure the reliability of the regression results in this paper, the following two methods is adopted for robustness test: one is to replace the explained variable, using GTFP measured by ML index as the explained variable, and the other is to utilize geographic and economic distance nested matrix (*W*4) to replace geographic distance matrix (*W*_2_). Table 10 reports robustness test results.

**Table 10:** The result of robustness and endogeneity test.

Variables	*ML*		*W4*		*GTFP*_*t-1*_	
	*Coef*.	*S*.*D*.	*Coef*.	*S*.*D*.	*Coef*.	*S*.*D*.
*GTFP*_*t-1*_					0.224***	0.019
*lnFDI*	0.125***	0.036	0.219***	0.031	0.089***	0.028
*FE*	-0.030	0.028	-0.019	0.025	-0.059***	0.021
*lnFDI×FE*	-0.068***	0.023	-0.098***	0.021	-0.025	0.018
*W×lnFDI*	0.513***	0.080	0.458***	0.074	0.330***	0.062
*W×FE*	-0.364***	0.067	-0.205***	0.052	-0.085*	0.051
*W×(LnFDI×FE)*	-0.347***	0.053	-0.354***	0.062	-0.188***	0.040
***LR_Direct***						
*lnFDI*	0.186***	0.036	0.285***	0.034	0.127***	0.027
*FE*	-0.071***	0.026	-0.044*	0.023	-0.068***	0.020
*lnFDI×FE*	-0.107***	0.025	-0.143***	0.024	-0.045**	0.018
***LR_Indirect***						
*lnFDI*	0.836***	0.118	1.078***	0.167	0.541***	0.086
*FE*	-0.560***	0.098	-0.409***	0.093	-0.157**	0.077
*lnFDI×FE*	-0.552***	0.079	-0.762***	0.135	-0.294***	0.060
***LR_Total***						
*lnFDI*	1.022***	0.127	1.363***	0.181	0.668***	0.096
*FE*	-0.631***	0.104	-0.453***	0.096	-0.225***	0.083
*lnFDI×FE*	-0.659***	0.091	-0.906***	0.148	-0.339***	0.069
***ρ***	0.392***	0.046	0.503***	0.408	0.395***	0.044
***Sigma2***	0.013***	0.001	0.011***	0.001	0.007***	0.001
***R***^***2***^	0.134		0.227		0.289	

It can be found in Table 10 that no matter which method is used, both the direct and indirect effects of FDI are positive and have passed the significance test at the 1% level, which indicates that FDI plays significant positive effects on the local and its neighboring GTFP; both the direct and indirect of fiscal expenditure are significantly negative at the 1% significance level, confirming that it exerts negative effects on GTFP in the local and its neighboring regions. In addition, both the direct and indirect effects of the interaction of FDI and fiscal expenditures are also significantly negative at the 1% significance level. This suggests that the interaction between FDI and fiscal expenditures hinders the local and its neighboring GTFP growth. To sum up, except for small-range fluctuations in the coefficients, the signs and significance of the core explanatory variable coefficients using the above two methods are basically consistent with the baseline regression, indicating that the results of the baseline regression are robust and reliable.

### 5.5 Endogeneity test

Generally speaking, there are three main sources of potential endogeneity: missing variables, simultaneity between explanatory variables and interpreted variables, and measurement errors. Considering that GTFP may have an "inertial effect",that is, the previous GTFP will affect the current GTFP [79]. To avoid the resulting error term reflecting a systematic pattern due to the "inertial effect", we introduce one period lag *GTFP* into Eq (8) [8]. In this way, the endogenous problems caused by the GTFP’s spatial lag, time lag, space-time lag, and missing variables are alleviated, and this enables us to extract the history of the other independent variables in the regression so that their inclusion represents the impact of new information. We obtains the dynamic SDM of Eq (18) and performs regression analysis, and the result are shown in Table 10.

$$\begin{array}{l} GTFP_{i,t}=\alpha_{0}+\alpha_{4}GTFP_{i,t-1}+\rho W_{i,t}\ln GTFP_{i,t}+\alpha_{1}\ln FDI_{i,t}+\alpha_{2}FE_{i,t}+\alpha_{3}\ln FDI_{i,t}\times FE_{i,t}\\ \;+\beta_{1}\ln HR_{i,t}+\beta_{2}\ln ER_{i,t}+\beta_{3}\ln PGDP_{i,t}+\beta_{4}\ln TD_{i,t}+\beta_{5}URBAN_{i,t}+\beta_{6}\ln TECH_{i,t}\\ \;+\theta_{1}W_{i,t}\ln FDI_{i,t}+\theta_{2}W_{i,t}FE_{i,t}+\theta_{3}W_{i,t}\ln FDI_{i,t}\times FE_{i,t}+\mu_{i}+\nu_{t}+\varepsilon_{it} \end{array}$$ (18)

It can be seen from Table 10 that the spatial lag coefficient ρ is significantly positive, and the direct, indirect and total effects of the lag-term of GTFP have all passed the 1% significance test, and the coefficient signs and significance levels of the core explanatory variables remain basically unchanged, which indicates that there is no endogenous problem in the baseline regression and its results are reliable. At the same time, the coefficients estimated by static panel and dynamic panel are consistent in size and sign, which further shows that the selection of variables is reasonable and the model is robust.

## 6. Conclusions and policy implication

Based on theoretical analysis, using the panel data of China’s mainland 30 provinces during 2003–2017, this paper empirically investigates the relationship between FDI, fiscal expenditure and GTFP by applying the spatial Durbin model, on basis of which regional heterogeneity analysis, robustness check and endogeneity test are conducted. The following findings can be drawn from this paper:

1. Through global and local spatial autocorrelation analysis, it is revealed that there exist significant spatial correlation and regional heterogeneity in FDI, fiscal expenditure and GTFP in China.
2. From the national perspective, FDI significantly promotes the local and its neighboring GTFP growth in China, the pollution heaven hypothesis is not supported here. Additionally, fiscal expenditure significantly hinders the growth of GTFP in the local and its neighboring regions, and it hampers GTFP growth by weakening the economic growth effect of FDI in China.
3. From the regional perspective, FDI significantly promotes the Eastern GTFP growth, while hinders the Central-Western GTFP growth. Fiscal expenditure has a significant negative effect on the Eastern GTFP, while exerts a significant positive effect on the Central-Western GTFP. The impacts of the interaction between FDI and fiscal expenditure on the Eastern and Central-Western GTFP are respectively negative and positive.

Based on above findings, to fully coordinate the relationship between FDI, fiscal expenditure and GTFP in China, we propose the following policy implications for policy makers.

1. China’s different regions should adjust the structure and scale of FDI based on their actual conditions to give fully play to their technological spillover effects, and determine reasonable environmental thresholds to attract the entry of high-quality FDI, find the optimal FDI structure and scale suitable for the growth of GTFP in the region. At the same time, it will exert its technology spillover effect across geographical boundaries and promote the spread and spread of its spatial spillover effect in a wider range, higher level, and deeper level, thereby effectively promoting the promotion of GTFP in other regions.
2. The Chinese central and local governments need to abandon the GDP-oriented economic development mode, and give environmental protection at a higher priority, as well as avoid the competition of “race to the bottom” between local governments. It is of great importance to achieve the coordination between economy, energy and environment.
3. The structure of fiscal expenditure needs to be further adjusted by the Chinese central and local governments, break the expenditure model of "emphasis on infrastructure and light people’s livelihood", more fiscal expenditure should be invested in environmental protection field, with the aim to reduced resources misallocation. At the same time, this effect will be radiated to neighboring areas to realize the spatial spillover of GTFP growth in neighboring areas, and thus promote China’s GTFP growth.
4. The Chinese local governments should actively respond to the policies and strategies of the central government in accordance with the differences in factor endowments across regions, and give full play to the synergy and benign interaction between FDI and fiscal expenditures, and achieve high-quality development in China. The eastern region should continue to be led by FDI, and on this basis, focus on adjusting the structure of fiscal expenditure, guiding the synergy and benign interaction between FDI and fiscal expenditure, and achieving a rapid increase in GTFP in the eastern region. The central and western regions need to further encourage the development of FDI, give full play to the role of FDI in promoting GTFP, and focus on the absorption and digestion of core technologies in the process of foreign capital introduction, so that FDI can play an economic driving effect. At the same time, continue to strengthen environmental fiscal expenditures, expand the scale of fiscal expenditures in education and technology, and provide a good technological environment for improving GTFP.

## Supporting information

S1 File (ZIP)
